# Positive side effects of Ca antagonists for osteoarthritic joints—results of an in vivo pilot study

**DOI:** 10.1186/s13018-014-0138-8

**Published:** 2015-01-09

**Authors:** Kiriakos Daniilidis, Philipp Georges, Carsten O Tibesku, Peter Prehm

**Affiliations:** Department of Orthopaedic Surgery, Annastift Hanover (Medical School Hanover; MHH), Anna-von-Borries-Strasse 1-7, 30625 Hannover, Germany; Radiologie am Theater, Neuer Platz 4, 33098 Paderborn, Germany; Sporthopaedicum Straubing/Regensburg, Bahnhofplatz 27, D-94315 Straubing, Germany; Institute for Physiological Chemistry, Medical Faculty University of Münster, Münster, Germany

**Keywords:** Osteoarthritis, Calcium antagonist, Cardiac arrhythmia, Cartilage

## Abstract

**Background:**

We have shown previously that some calcium antagonists inhibit hyaluronan export, loss of proteoglycans, and degradation of collagen from osteoarthritic cartilage. Clinically approved calcium antagonists normally are prescribed for cardiac arrhythmia. In the present study, we compared the effect of these drugs on osteoarthritic patients which had received no medication and patients which were also diagnosed for cardiac arrhythmias and were treated with calcium antagonists. The effects and the side effects of the used drugs were analyzed.

**Method:**

We used the Lequesne questionnaire to examine patients with osteoarthritis (212 patients, control group receiving no calcium antagonists) and patients with cardiac arrhythmia and osteoarthritis (188 patients treated with various calcium antagonists). The answers of the questionnaires were transformed into the Lequesne scoring system quantifying the severity of the disease. The Lequesne score is a standardized questionnaire focused on osteoarthritis. It is a 24-scale questionary in which low scores indicate low functional activity.

**Results:**

The data showed that the mean score of the control group (6.2) was higher than the treated group (5.2), the drugs differed in their efficiency. Verapamil had a slightly worse score and Azupamil, Escor, Felodipine, and Nifedipine showed no alteration. Adalat, Amlodipine, Carmen, Nitrendipin, and Norvasc lead to an improvement.

**Conclusion:**

These results suggest that inhibition of hyaluronan export may have a beneficial effect on human osteoarthritis.

**Electronic supplementary material:**

The online version of this article (doi:10.1186/s13018-014-0138-8) contains supplementary material, which is available to authorized users.

## Background

Osteoarthritis (OA) is the most common specific joint disease in humans and the largest cause of chronic disability in the elderly [1]. It occurs when the equilibrium between breakdown and repair of the joint tissues becomes unbalanced. There are currently no pharmacological interventions available to patients for modifying the underlying disease in spite of intensive research over the past five decades [2,3]. Previously, it was thought that proteolytic degradation of collagen and aggrecan was primarily responsible for cartilage breakdown. Efforts to develop protease inhibitors led to compounds that were chondroprotective *in vitro* and in animal models, but results from clinical trials were equivocal [4]. However, increased hyaluronan synthesis precedes the stimulation of protease synthesis [5,6]. When we discovered that hyaluronan was exported by the multidrug resistant protein MRP5, a whole series of hyaluronan export inhibitors were suddenly available that we consequently tested for their effects on osteoarthritic reactions of chondrocytes in culture, on bovine cartilage explants, and in a rat model of osteoarthritis [7-10]. The hyaluronan export inhibitors tested effectively reduced not only hyaluronan export but also subsequent osteoarthritic reactions such as proteoglycan loss and collagen degradation. Some of the drugs were approved calcium antagonists prescribed for cardiac arrhythmia.

Since new drug development is a tedious and cumbersome process, we analyzed here the potential of several drugs to ameliorate the symptoms of osteoarthritis in patients which suffered from cardiac arrhythmias and were treated with the calcium antagonists and compared the data with patients which solely had osteoarthritis.

The study was carried out in accordance with the World Medical Association Declaration of Helsinki.

## Materials and methods

The Lequesne questionnaire [11] was modified to incorporate the patient’s weight and height. The Lequesne score is a standardized questionnaire focused on osteoarthritis. It is a 24-scale questionary in which low scores indicate low functional activity (Table 1).

**Table 1:** **Lequesne score**

**Drugs**	**Treated patients (mean score)**	**Number of patients**	**SD**	***p*** **value**
Adalat® (Nifedipine)	4.0	5	3	0
Amlodipine® (Amlodipine)	3.5	22	2.97	0
Azupamil® (Verapamil)	6	8	3.59	0
Carmen® (Lercanidipine)	3	21	2.91	0
Escor® (Nilvadipine)	6	5	4.72	0
Felodipine® (Felodipine)	5	7	4.1	0
Nifedipine® (Nifedipine)	6.2	15	5.21	0
Nitrendipine® (Nitrendipin)	2.1	8	2.8	0
Norvasc® (Amlodipine)	4.8	48	5	0
Verapamil® (Verapamil)	6.9	49	5	0
Control group	6.2	212	4.85	0

*SD* standard deviation.

It was answered by 400 patients with osteoarthritis (207 women and 193 men). More than 99% of the patients were older than 50 years. Both the control and the active treatment groups have been diagnosed for osteoarthritis for more than 1 year before and the active treatment group has received calcium antagonists for more than 1 year.

Pre-study calculations revealed that 198 patients for each group were required to arrive at a statistical significance of *p* < 0.05 and a power of 80% for a difference in one unit in the Lequesne score. Matched pairs were established for potential interference variables such as gender, age, and body mass index. The first evaluations of equivalences were performed stepwise with 100, 200, 300, and 400 patients. Finally, a complete equivalence could not be achieved for gender (55% women in the active treatment group and 45% women in the control group) and body mass index (76.27 ± 9.1 kg in the control group).

The Lequesne score correlates significantly with pain and consists of three subscores which were calculated individually and together: pain and discomfort, maximum distance walked, and activities of daily living with a maximum score of 8 for each subscore and a total score of 24 (see Additional file 1). A difference in one score unit is regarded as clinically relevant. The groups were evaluated by the Levene test for equality of variance and by the *T*-test for equality of the mean.

## Results

In addition to the calcium (Ca) antagonists analyzed previously for inhibition of hyaluronan export, we included here those compounds that were currently used as medications. Furthermore, we found a slight drift concerning the inhibitory concentrations for hyaluronan which were in the micromolar range, whereas the Ca antagonists were supposed not to inhibit hyaluronan through alterations of the intracellular Ca^2+^ concentration but rather by direct interaction with the hyaluronan exporter MRP5.

### Correlation of export inhibition and statistics

The total Lequesne score and the three subscores were calculated for 188 patients in the active treatment group and 212 patients in the control group (Table 2). The total Lequesne score showed a significant difference of the active treatment group versus the control group. This significance was mainly found in subgroups 2 and 3 for the maximum walking distance and activities of daily living, respectively, whereas the difference in subgroup 1 for pain and discomfort was insignificant.

**Table 2:** **Total and subgroup Lequesne score**

**Group**	**Mean**	**SD**	***p*** **value**
Total			
Active treatment	5.23	1.89	0.044
Control	6.19	1.57	
Subgroup 1			
Active treatment	1.64	1.73	0.607
Control	1.73	1.84	
Subgroup 2			
Active treatment	1.72	1.84	0.044
Control	2.08	2.06	
Subgroup 3			
Active treatment	1.87	5.23	0.008
Control	2.38	6.19	

*SD* standard deviation.

The significance was also calculated for the individual drugs versus the control group. Only those groups with five or more patients were incorporated. Significant amelioration of the Lequesne score was found for Amlodipine®, Carmen®, and Nitrendipine®. It is noteworthy that the difference for the amlodipine-containing drug Norvasc® was not significant.

## Discussion

The present paper describes that Ca antagonists inhibit hyaluronan export by MRP5 which is the principle hyaluronan exporter for fibroblasts and chondrocytes and they simultaneously improved the Lequesne score for osteoarthritis. There were gross differences in their activities. Since the effective concentrations as Ca antagonists were order of magnitude lower that the inhibitory concentration for hyaluronan export inhibition, the beneficial effect on human osteoarthritis could not be mediated by direct with the exporter, because the high concentration for hyaluronan export inhibition will never be reached by drug treatment. Therefore, the action must rely in another yet unknown mechanism.

If the Ca antagonists give rise to such diverse responses in the Lequesne score as found in our results, it cannot rely on a common biochemical mechanism of modulating the Ca concentration. The responses must thus be attributed to the special structure of the individual compounds.

It was calculated initially that a patient number of 198 is required to allow a statistically significant conclusion. This calculation should also apply for the individual compounds. However, the large deviations of some compounds in the Lequesne score from the control were based on less than 10 patients. Thus, this figure should not be regarded as significant.

Although the results for single drugs were not significant, the overall effect of Ca antagonists on OA patients as compared to controls may nevertheless indicate an underlying biochemical mechanism. We recently discovered that hyaluronan export by MRP5 is dependent on concurrent K^+^ efflux through Kir channels [12].

The Kir channels belong to the calcium-activated K^+^ channels [13]. Thus, blockage of calcium entry into the cytoplasm may inhibit K^+^ efflux and, simultaneously, also excessive hyaluronan export. If this scenario is valid, treatment of OA patients with hyaluronan-inhibiting drugs may be beneficial.

There are several limitations to the current analysis that must be taken into account when analyzing these results. Firstly, although a control arm was offered as means of a comparison, there were notable differences in the demographics between the groups. The fact that we deal with a heterogenous group of Ca antagonists leads to an unknown range of bias. It is possible that these factors influenced the overall results. The fact that we did not perform a power analysis and that we did not randomize the patients should be stated as a major limitation of the present study. Furthermore, the present study deals with patients who suffered on OA; that means that they could suffer also from pain caused by other joints which could be affected. Another reason could be that the patients were affected positive or negative by other co-medication.

## Conclusion

Taken together, the presented results indicate that Ca antagonists might be efficient in preventing progression of osteoarthritis. However, in the absence of randomized clinical trial data, the use of the drug cannot be justified yet. Hence, data from a randomized study are needed to better elucidate the potential of Ca antagonists in preventing progression of osteoarthritis.

## Additional file

Additional file 1:
**Index of Severity for Osteoarthritis of the Knee by Lequesne et al. **Lequesne et al. developed an index of severity for osteoarthritis for the knee (ISK). This can be used to assess the effectiveness of therapeutic interventions.
