# Supplementary material for: Positive side effects of Ca antagonists for osteoarthritic joints—results of an in vivo pilot study
Source: J Orthop Surg Res. 2015 Jan 9;10:1. doi: 10.1186/s13018-014-0138-8 (PMC4296554; doi:10.1186/s13018-014-0138-8)
Supplement: Additional file 1: — Index of Severity for Osteoarthritis of the Knee by Lequesne et al. Lequesne et al. developed an index of severity for osteoarthritis for the knee (ISK). This can be used to assess the effectiveness of therapeutic interventions. [file 13018_2014_138_MOESM1_ESM.pdf]

Index of Severity for Osteoarthritis of the Knee by Lequesne et al

Overview:

Lequesne et al developed an index of severity for osteoarthritis for the knee (ISK). This can be used to assess the effectiveness of therapeutic interventions.

Sections for index:

(1) pain or discomfort

(2) maximum distance walked

(3) activities of daily living

I Pain or Discomfort

Parameter	Finding	Points
pain or discomfort during nocturnal bedrest	none	0
	only on movement or in certain positions	1
	without movement	2
duration of morning stiffness or pain after getting up	none	0
	< 15 minutes	1
	>= 15 minutes	2
remaining standing for 30 minutes increases pain	no	0
	yes	1
pain on walking	none	0
	only after walking some distance	1
	early after starting	2
pain or discomfort after getting up from sitting without use of arms	no	0
	yes	1

where:

- A change in a 1991 version was to have the duration of morning stiffness scored 0 if it was 1 minute or less and 1 if it was from 1 to less than 15 minutes.

- Pain on walking in a 1991 version expanded "early after starting" to "after initial ambulation and increasingly with continued ambulation"

II. Maximum Distance Walked

Parameter	Finding	Points
maximum distance walked	unlimited	0
	> 1 kilometer but limited	1
	about 1 kilometer (about 15 minutes)	2
	about 500 - 900 meters (about 8-15 minutes)	3
	from 300 - 500 meters	4
	from 100 - 300 meters	5
	< 100 meters	6
walking aids required	None	0
	1 walking stick or crutch	1
	2 walking sticks or crutches	2

III. Activities of Daily Living

Parameter	Finding	Points
able to climb up a standard flight of stairs	easily	0
	with mild difficulty	0.5
	with moderate difficulty	1.0
	with marked difficulty	1.5
	impossible	2.0
able to clumb down a standard flight of stairs	easily	0
	with mild difficulty	0.5
	with moderate difficulty	1.0
	with marked difficulty	1.5
	impossible	2.0
able to squat or bend at the knee	easily	0
	with mild difficulty	0.5
	with moderate difficulty	1.0
	with marked difficulty	1.5
	impossible	2.0
able to walk on uneven ground	easily	0

	with mild difficulty	0.5
	with moderate difficulty	1.0
	with marked difficulty	1.5
	impossible	2.0

index of severity =

= SUM(points for all parameters)

Interpretation:

• minimum points for each section: 0

• maximum points for each section: 8

• minimum index score: 0

• maximum index score: 24

Index Score	Handicap
0	none
1 - 4	mild
5 - 7	moderate
8 - 10	severe
11 - 13	very severe
>= 14	extremely severe

Modifications

The index was modified in 1997 with some minor changes to morning stiffnes and termed the "algofunctional index".

References:

Lequesne M Mery C et al. Indexes of severity for osteoarthritis of the hip and knee. Scand J Rheumatology. 1987; Supplement 65: 85-89.

Lequesne M. Indices of severity and disease activity for osteoarthritis. Seminars in Arthritis and Rheumatism. 1991; 20 (supplement 2): 48-54.

Lequesne MG. The algofunctional indices for hip and knee osteoarthritis. J Rheumatol. 1997; 24: 779-781.
